# Associations of angiopoietin-like protein 7 with coronary collateral circulation and prognosis of patients with severe coronary artery stenosis

**DOI:** 10.3389/fcvm.2025.1559267

**Published:** 2025-10-10

**Authors:** Congyi Cheng, Chunlin Wang, Weichun Zhu, Cong Su, Youran Dong, Junlin Teng, Kang Fu, Chungang Zhai, Lei Qiao, Wenqiang Chen

**Affiliations:** ^1^ State Key Laboratory for Innovation and Transformation of Luobing Theory; Key Laboratory of Cardiovascular Remodeling and Function Research of MOE, NHC, CAMS and Shandong Province, Department of Cardiology, Qilu Hospital of Shandong University, Jinan, China; ^2^ Department of Cardiology (Chest Pain Center), Central Hospital Affiliated to Shandong First Medical University, Jinan, China

**Keywords:** coronary heart disease, angiopoietin-like protein 7, coronary collateral circulation, severe coronary stenosis, clinical prognosis, angiogenesis

## Abstract

**Background:**

Angiogenesis and coronary collateral circulation (CCC) formation promote cardiac repair following severe coronary stenosis (SCS) or myocardial infarction (MI). Angiopoietin-like protein 7 (ANGPTL7) is a secreted protein associated with angiogenesis, but its role in CCC formation remains unclear.

**Objective:**

The aim of this study was to investigate the role of ANGPTL7 in angiogenesis and evaluate the predictive value of serum ANGPTL7 in CCC formation and the prognosis of patients with SCS.

**Materials and methods:**

The RNA sequencing was performed on myocardial tissues of mice to analyze the alterations of angiogenesis-related genes after MI. 100 patients with angiographically proven SCS and 36 controls were enrolled and retrospectively followed up. Serum ANGPTL7 was measured by enzyme-linked immunosorbent assays (ELISA). Human umbilical vein endothelial cells (HUVECs) and exogenous human recombinant ANGPTL7 protein were applied in assays including CCK-8, scratch, tube formation, cell immunofluorescence and western blot to demonstrate the proangiogenic effect of ANGPTL7. Gene Set Enrichment Analysis (GSEA) was used to perform KEGG pathway enrichment analysis on downstream mechanisms by which ANGPTL7 promoted angiogenesis.

**Results:**

The transcriptional level of *Angptl7* was upregulated in ischemic myocardial tissues of MI mice, and its serum levels increased in both mice post-MI and patients with SCS. Spearman correlation analysis indicated that serum ANGPTL7 levels were positively correlated to CCC grades (*r* = 0.518, *P* < 0.001). Kaplan–Meier curves showed a higher serum ANGPTL7 was associated with a lower incidence of major adverse cardiovascular events (MACE) in patients with SCS (Log-rank test, *P* = 0.002). Cox proportional hazards regression analyses showed that serum ANGPTL7 level was remained a protective factor after adjusting for different covariates. Time-dependent receiver-operating characteristics (ROC) curves further explored the prognostic value of ANGPTL7, with the area under the curve (AUC) of 0.77 at 1 year, 0.70 at 2 years and 0.85 at 3 years. Additionally, ANGPTL7 enhanced endothelial cell proliferation, migration and capillary-like structure formation, indicating a proangiogenic effect *in vitro*.

**Conclusion:**

ANGPTL7 serves as a predictive biomarker for CCC levels and the prognosis of patients with SCS, which probably attributed to its proangiogenic properties.

## Graphical Abstract

**Figure:**
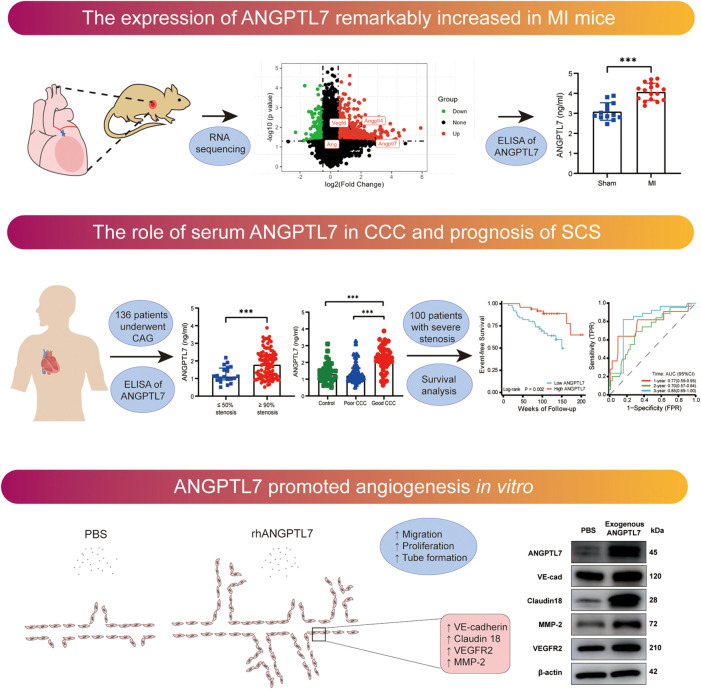

## 1 Introduction

Coronary heart disease (CHD) is the leading cause of death in both developed and developing countries nowadays (1). The formation of atherosclerotic plaques in epicardial arteries causes coronary luminal stenosis and myocardial ischemia (2). However, certain patients develop coronary collateral circulation (CCC) as the compensatory mechanism to improve the myocardial blood supply, thereby resulting in a lower incidence of poor prognosis (3). Recent studies have demonstrated that angiogenesis plays a critical role in the formation of CCC, especially at the early stage (4).

Angiopoietin-like (ANGPTL) proteins are a family of secreted glycoproteins structurally similar to the angiopoietins, consist of ANGPTL1-8. ANGPTL proteins are widely expressed in various tissues and involved in many pathophysiological processes such as angiogenesis, inflammation, lipid metabolism and development of cancer (5). Among these functions, the primary role of ANGPTLs is to regulate angiogenesis (6, 7). For example, ANGPTL1 has been defined as an anti-angiogenic molecule (8), while ANGPTL2, 3 and 6 positively regulate endothelial cell vasculogenesis (9–11). ANGPTL4 may be pro- or anti-angiogenic as the microenvironment changes (12, 13).

Angiopoietin-like protein 7 (ANGPTL7), also called cornea-derived transcript 6 or AngX, was first discovered in human cornea in 1998 (14). As a member of the angiopoietin-like factor family, the current understanding about the physiological functions of ANGPTL7 is still limited. There have been relatively few studies about ANGPTL7 up till now, mainly focusing on inflammation and angiogenesis (15–17). Furthermore, the role of ANGPTL7 in angiogenesis is still controversial (16, 18). ANGPTL7 was initially believed to be a negative regulator of angiogenesis due to it's specific presence in the cornea tissue (18). But recent studies have revealed that ANGPTL7 promotes angiogenesis and cancer progression (16).

In terms of myocardial ischemia-induced angiogenesis after coronary stenosis, the role of ANGPTL7 is still unknown. In this study, we aim to investigate the role of ANGPTL7 in angiogenesis and evaluate the predictive value of serum ANGPTL7 in CCC formation and the clinical prognosis of CHD patients with severe coronary stenosis (SCS).

## 2 Materials and methods

### 2.1 Clinical study

The single-center retrospective cohort study complied with the principles of the 1975 Declaration of Helsinki and received approval from the Ethics Review Committee of Qilu Hospital of Shandong University (Approval No.2020042). Informed consent was obtained from the participants.

#### 2.1.1 Participants

**Figure 1:**
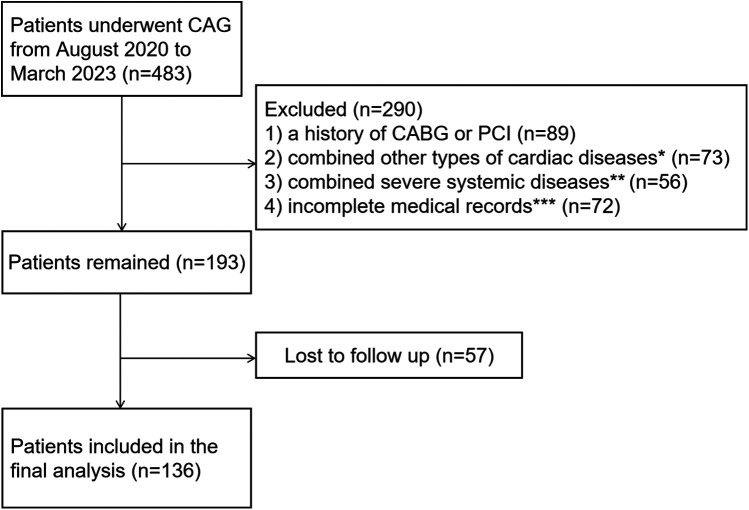
Flow chart of patient enrollment. *Including severe arrhythmias, congenital heart disease, valvular heart disease and non-ischemic cardiomyopathy. **Including severe coagulopathy, hypothyroidism or hyperthyroidism, severe renal or hepatic disease, malignant tumors and autoimmune disease. ***Including 38 patients without serum samples, 14 patients without detailed coronary angiography images, and 20 patients without necessary auxiliary examination results, such as blood routine, blood biochemical tests, echocardiography et al.

The study initially included an aggregate of 483 hospitalized patients who underwent coronary angiography (CAG) for the first time at Qilu Hospital of Shandong University between August 2020 and March 2023. Considering the potential involvement of ANGPTL7 in other diseases, patients with severe coagulopathy (*n* = 4), thyroid dysfunction (hypothyroidism or hyperthyroidism, *n* = 7), severe renal or hepatic disease (serum creatinine >134umol/L or alanine aminotransferase ≥3×upper normal value, *n* = 30), malignant tumors (*n* = 6), autoimmune diseases (*n* = 9), other types of cardiac diseases (including severe arrhythmias, congenital heart disease, valvular heart disease, and non-ischemic cardiomyopathy, *n* = 73), a history of coronary artery bypass grafting (CABG) or percutaneous coronary intervention (PCI) (*n* = 89), as well as those with incomplete medical records (*n* = 72) were excluded. Of the remaining 193 patients, 55 patients with <90% stenosis in all three main coronary arteries served as the control group, while 138 patients with SCS—defined as luminal stenosis ≥90% in at least one main coronary artery [left anterior descending artery (LAD), left circumflex artery (LCX) or right coronary artery (RCA)], constituted the study group. These participants were followed up during August 2024 by telephone for 13–207 weeks post-discharge: 100 (72.46%) patients with SCS and 36 (65.45%) in the control group finally completed the telephone follow-up (Figure 1). To evaluate the possibly adverse effects on the reliability of the results caused by the inclusion/exclusion process, differences in baseline characteristics between patients who were lost to follow-up and those who were retained were analyzed (Supplementary Tables 1, 2).

#### 2.1.2 Data collection and definitions

Detailed clinical data was meticulously gathered from medical records by well-trained clinicians who were blinded to the study protocol. The data encompassed general conditions [age, gender, body mass index (BMI), hypertension, diabetes mellitus (DM), smoking, alcohol consumption, family history of coronary heart disease (FH-CHD) and carotid atherosclerostic plaques], laboratory tests [N-terminal pro-brain natriuretic peptide (NT-proBNP), high-sensitivity cardiac troponin I (hs-cTNI), serum creatinine (SCr), alanine aminotransferase (ALT), aspartate transaminase (AST), fasting plasma glucose (FPG), total cholesterol (TC), low-density lipoprotein cholesterol (LDL-C), high-density lipoprotein cholesterol (HDL-C) and triglyceride (TG)], echocardiographic assessment [left ventricular ejection fraction (LVEF), intraventricular septum (IVS) and segmental wall-motion abnormality], severity of CAG [coronary arteries with severe stenosis (≥90%) and multivessel disease], information on cardiovascular medications [antiplatelet drugs, statins, beta-blockers, angiotensin-converting enzyme inhibitors (ACEI)/angiotensin receptor blockers (ARB)/angiotensin receptor & neprilysin inhibitors (ARNI), calcium channel blockers (CCB), nitrates and nicorandil] and the acceptance of coronary revascularization threpy (PCI or CABG). Peripheral venous blood samples were collected early in the morning following an overnight fasting and the routine laboratory parameters above were measured. BMI was calculated as weight (kg)/the square of height (m^2^). Hypertension was diagnosed in accordance with the 2018 ESC/ESH Guideline (19). DM was defned as FPG ≥7.0 mmol/L, random blood glucose ≥11.1 mmol/L, 2 h plasma glucose after oral glucose tolerance test (OGTT) ≥ 11.1 mmol/L, or using insulin or oral hypoglycemic agents (20). FH-CHD was defned as the occurrence of CHD in any immediate relative within three generations.

#### 2.1.3 Coronary angiography

Coronary angiography was performed through either the radial or the femoral approach. The angiographic images were analyzed by two experienced cardiologists who adhered to the lesion classification scheme of the American College of Cardiology/American Heart Association (21). CCC was assessed in patients who had a diameter stenosis of ≥90% in at least one major coronary artery (LAD, LCX or RCA). The level of collateral circulation were quantified using the Rentrop score system (22) as follows: 0 = none, 1 = filling of side branches only, 2 = partial filling of the epicardial segment, and 3 = complete filling of the epicardial segment. The two cardiologists who reviewed the angiograms were blinded to the study's objective, with any disagreements being resolved through consensus with a third reviewer. Patients (*n* = 100) were categorized into the poor CCC group (grade 0 or 1, *n* = 48) and the good CCC group (grade 2 or 3, *n* = 52) based on the Rentrop score.

#### 2.1.4 Serum ANGPTL7 measurement of patients

Blood samples from all the included patients were obtained after an overnight fast at 8:00 AM prior to CAG. Within one hour of collection, the samples were immediately centrifuged at 3,000 r/min for 15 min at 4°C to obtain serum. Subsequently, the serum samples were collected in 1.5 ml Eppendorf (EP) tubes and stored at −80°C until analysis. The levels of ANGPTL7 in the serum were measured using an enzyme-linked immunosorbent assay (ELISA) kit (Human ANGPTL7 ELISA kits; Thermo Fisher; EH30RBX5).

#### 2.1.5 Outcome

The primary major adverse cardiovascular event (MACE) endpoint of our clinical study was defined as the composite of all-cause death (both cardiovascular and non-cardiovascular death), non-fatal myocardial infarction (MI), non-fatal stroke and coronary artery revascularization (PCI or CABG).

### 2.2 Mouse and *in vitro* study

#### 2.2.1 Mouse model construction

All experimental procedures involving animals were conducted under the approval of the Ethics Committee of Qilu Hospital of Shandong University [KYLL-2022(ZM)-225].

Male C57BL/6J mice, aged 8 weeks and weighing between 22 and 28 g, were sourced from Beijing Weitong Lihua Experimental Animal Technology Corporation (Beijing, China). Following a 1-week acclimation period on standard mouse chow, the mice were randomly allocated into either a model group or a sham-operated group. The MI model was induced by the ligation of left coronary artery, whereas the sham-operated group underwent an identical surgical procedure without coronary artery ligation. Four days post-MI, some of the mice were sacrificed and the myocardial tissues were collected for RNA sequencing.

#### 2.2.2 Serum ANGPTL7 measurement of mice

To collect blood samples (0.6–1.0 ml) from MI mice, left ventricular puncture was performed 4 days post-MI. Within 30 min post-collection, the blood samples were centrifuged at 3,000 r/min for 15 min at 4°C to separate the serum. The isolated serum samples were then transferred into 1.5 ml EP tubes and stored at −80°C until analysis. Serum ANGPTL7 levels were measured using a Mouse ELISA kit (LunChangShuo Biotech; ED-23368).

#### 2.2.3 Cell culture

Human umbilical vein endothelial cells (HUVECs) were obtained from Procell Life Science&Technology Company (Wuhan,China). These cells were cultured in endothelial cell medium (ScienCell, 1001) and maintained under standard conditions (37°C, 5% CO2) of a humidified incubator. For the purposes of our study, the HUVECs were utilized in the CCK8 assay, scratch assay, tube formation assay, immunofluorescence assay and western blot (23).

#### 2.2.4 CCK-8 assay

HUVEC proliferation was analyzed using the enhanced cell counting kit-8 (CCK-8, Beyotime, C0038). Briefly, cell suspensions (5.0 × 103 cells in 100 µl per well) were initially pre-cultured in 96-well plates for 24 h. For the experimental group, 1 µl of human recombinant ANGPTL7 protein (R&D Systems, 914-AN) was added to each well, while in the control group, an equivalent volume of phosphate-buffered saline (PBS) was added. Following incubation for an additional 24 h, the CCK-8 solution (10 µl per well) was introduced and the cells were cultured for abother 4 h at 37°C. Absorbance was subsequently measured at 450 nm using a multi-functional microplate reader (Bio Tek, USA).

#### 2.2.5 Scratch assay

Cell suspensions (6.0 × 105 cells in 2 ml per well) were pre-cultured in 6-well plates for 24 h at 37°C in a 5% CO2 incubator. HUVECs were grown to confluence, after which monolayers were scratched using a sterile 200-μl pipette tip. Subsequently, the culture medium was completely removed and replaced with 1 ml of the appropriate serum-free endothelial cell medium (ECM) per well. For the experimental group, 10 µl of human recombinant ANGPTL7 protein (R&D Systems, 914-AN) was added to each well, while the control group received 10 µl of PBS. The scratch width in each well was observed, photographed, and recorded under an inverted microscope (Nikon, Japan, Eclipse Ti-S) at 0 and 24 h. ImageJ software was utilized to calculate the cell-free area within the scratch.

#### 2.2.6 Tube formation assay

To commence the experiment, Matrigel (Corning, 356234) was thawed overnight in a 4°C refrigerator. Subsequently, 50 µl of Matrigel was added to each well of a 96-well plate, which was then incubated at 37°C for 30 min to allow the Matrigel to rewarm. HUVEC suspensions, containing 2.0 × 104 cells in 100 µl per well, were prepared. The experimental group was treated with 1 µl of human recombinant ANGPTL7 protein (R&D Systems, 914-AN), whereas the control group received 1 µl of PBS. Both groups were incubated at 37°C in a 5% CO2 environment for 4 h. Observation of the samples was conducted using an inverted microscope (Nikon, Japan, Eclipse Ti-S) at 10× magnification. Photographs were taken to document the results, and ImageJ software was employed to quantify the number of branch points and analyze the level of tube formation.

#### 2.2.7 Cell immunofluorescence assay

Vascular endothelial cadherin (VE-cadherin), the principal component of endothelial adherens junctions, is essential for vessel maturation and stability and is frequently utilized in cell immunofluorescence assays (24). In this experiment, HUVECs grown on climbing slices were treated with either PBS or exogenous human recombinant ANGPTL7 protein (R&D Systems, 914-AN) for 24 h. Post-treatment, the cells were fixed with 4% paraformaldehyde for 15 min, followed by permeabilization with 0.4% Triton X-100 (Solarbio, T8200) for 15 min. Subsequently, the cells were blocked using 5% donkey serum at room temperature for 30 min. The cell slices were then incubated with rabbit anti-VE cadherin antibodies (Abcam, ab33168) for 3 h, followed by 1-h incubation with 1:200-diluted donkey anti-rabbit IgG H&L (Alexa Fluor® 488, Abcam, ab150061) while protected from light. Afterward, the cover glass with adhered cells was mounted on a slide prepped with an anti-fluorescence quenching agent containing DAPI (Beyotime, C1006). The cell climbing slices were subsequently observed and photographed under a fluorescence microscope (Nikon, Japan, DS-Ri2) and ImageJ software was used to quantify the fluorescence intensity.

#### 2.2.8 Western blot

HUVECs in the experimental group were treated with exogenous human recombinant ANGPTL7 protein (R&D Systems, 914-AN) at a final concentration of 1 µg/ml for 24 h, whereas cells in the control group received an equivalent volume of PBS. To obtain whole-cell proteins, HUVECs in these two groups were collected respectively and lysed on ice by RIPA lysis buffer (Solarbio, R0010) supplemented with phenylmethanesulfonyl fluoride (PMSF) (Solarbio, P0100). The whole-cell protein extracts were boiled for 10 min at 99°C and cooled on ice. Protein samples were separated on a 10% SDS-polyacrylamide gel (Epizyme Biotech, PG112-0) and transferred to a polyvinylidene difluoride (PVDF) membrane (Millipore, IPVH00010). The sheet was blocked with 5% dried skimmed milk solution for 2 h at room temperature and incubated overnight at 4°C with the appropriate western blot. Primary antibody dilutions included rabbit anti-ANGPTL7 antibody (Proteintech, 10396-1-AP), rabbit anti-VE-cadherin antibody (Abcam, ab33168), rabbit anti-Claudin18 antibody (Abcam, Ab203563), rabbit anti-MMP-2 antibody (CST, 87809S), rabbit anti-VEGFR2 antibody (CST, 2479S) and rabbit anti-β-actin antibody (CST, #4970). Thereafter, the PVDF membranes were washed with TBST (tris-buffered saline-Tween-20) three times and incubated with secondary antibodies (Zhongshan-Golden Bridge Biological Technology, ZB2301) for 1 h at room temperature. Finally, blots were visualized with enhanced chemiluminescence fluid and ImageJ software was used to analyze the protein relative gray values.

### 2.3 Bioinformatics analysis

Download GSE66360, an expression profiling dataset, from the Gene Expression Omnibus (GEO) database. The gene expression levels were determined using microarray analysis of enriched circulating endothelial cells isolated from patients with acute myocardial infarction. The patients were grouped according to the expression levels of ANGPTL7. KEGG pathway enrichment analysis was performed using Gene Set Enrichment Analysis (GSEA) software 4.4.0 between high and low ANGPTL7 expression data sets. |Normalized corrected ES values| (|NES|) >1, false discovery rate (FDR) <0.25 and adjusted *P*-value < 0.05 were considered as significance.

### 2.4 Statistical analysis

Statistical analysis was performed using SPSS 26.0 and R software 4.3.1. Shapiro–Wilk normality test was used to assess normality of continuous data. Continuous variables were presented as mean ± standard deviation (SD) or median with interquartile range, as appropriate. Differences among groups were compared using *t*-test or ANOVA test in case of normally distribution, and Mann–Whitney *U* test or Kruskal–Wallis *H* test in case of non-normally distribution. Categorical variables were presented as frequency counts with proportions and compared using the chi-square test. The associations between the serum ANGPTL7 levels and CCC grades or clinical prognosis were assessed using Spearman correlation analysis. ROC curves were plotted to evaluate the diagnostic or predictive value. Cox proportional hazards regression analyses were used to explore associations between serum ANGPTL7 level and MACE: Model 1 was unadjusted; Model 2 adjusted for age and gender; Model 3 adjusted for the same variables as Model 2 and all the variables significant on univariate analysis, including Rentrop score, LAD and LCX severe stenosis, hypertension and NT-proBNP; Model 4 adjusted for the same variables as Model 3, as well as DM, LDL-C, wall-motion abnormality and the usage of β-blocker and ACEI/ARB/ARNI, which were selected as clinically meaningful indicators with collinearity controlled (VIF < 5). Kaplan–Meier survival analysis was performed and the signifcance was assessed by Log-rank tests. A *P*-value of less than 0.05 was regarded as statistically significant.

## 3 Results

### 3.1 ANGPTL7 was upregulated in ischemic myocardial tissues of MI mice

**Figure 2:**
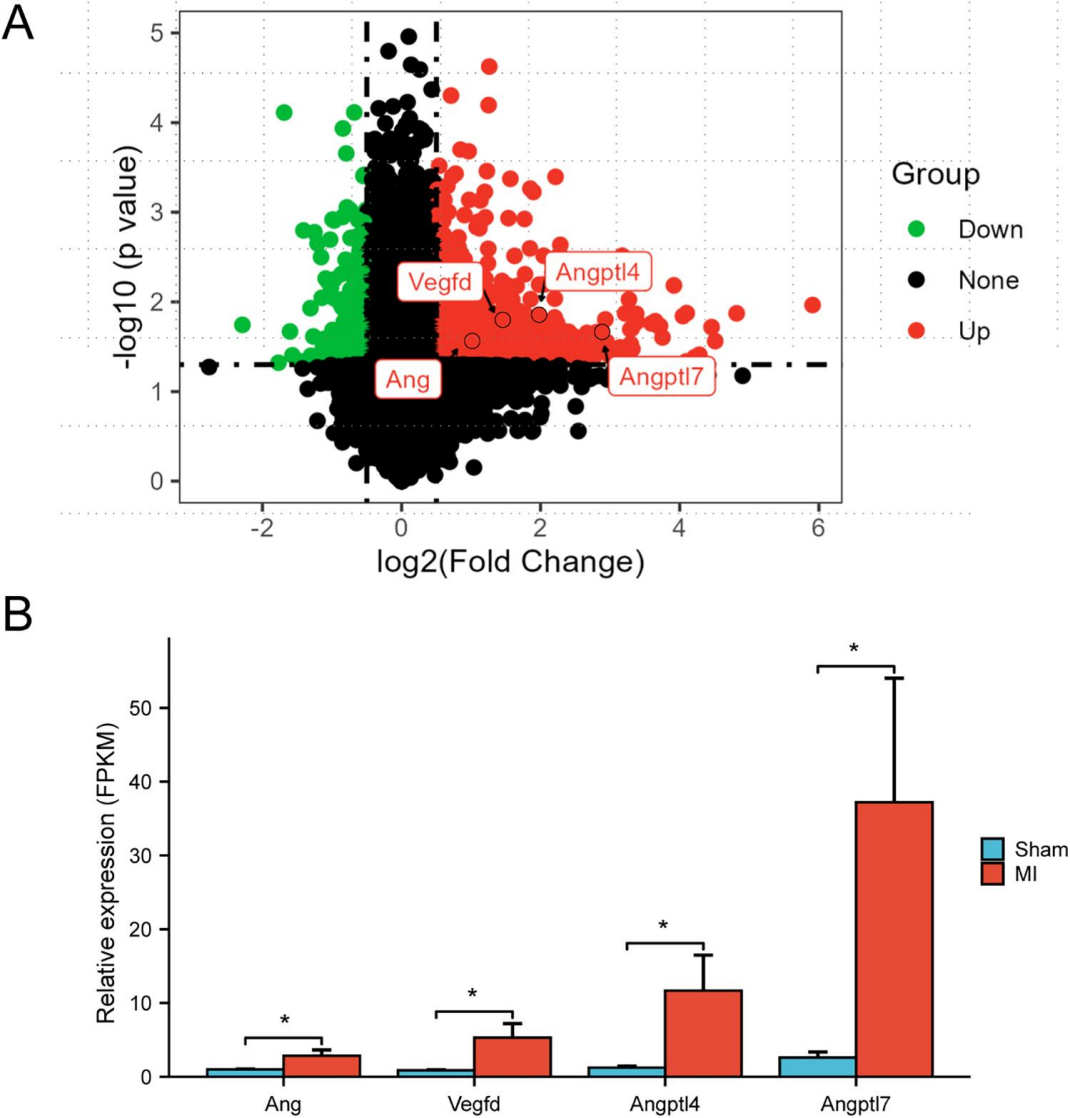
Expressions of four angiogenesis-related genes post-MI. **(A)** Volcano plot showing log2 fold-change of total gene expression in MI mice 4 days post-MI, compared with sham-operated mice. Genes that were significantly increased in MI group (*n* = 3) compared with sham group (*n* = 4) are highlighted on red, while those decreased are highlighted in green. **(B)** *Ang*, *Vegfd*, *Angptl4* and *Angptl7* were four angiogenesis-related upregulated genes (*P < 0.05). Bar graph revealed that *Angptl7* was the most significantly upregulated member among the four genes.

To analyze the alterations of genes related to angiogenesis after MI, the RNA sequencing was performed on myocardial tissues obtained from mice 4 days post-MI. Analysis of the RNAseq data through volcano plots and bar graphs uncovered an upregulation of four angiogenesis-related genes, namely *Ang*, *Vegfd*, *Angptl4* and *Angptl7*, in the ischemic myocardial tissues of MI mice. Among these genes, *Angptl7* exhibited the most significant elevation and was consequently selected for further investigation (Figures 2A,B).

### 3.2 Serum ANGPTL7 level was intimately associated with CCC of CHD patients

**Figure 3:**
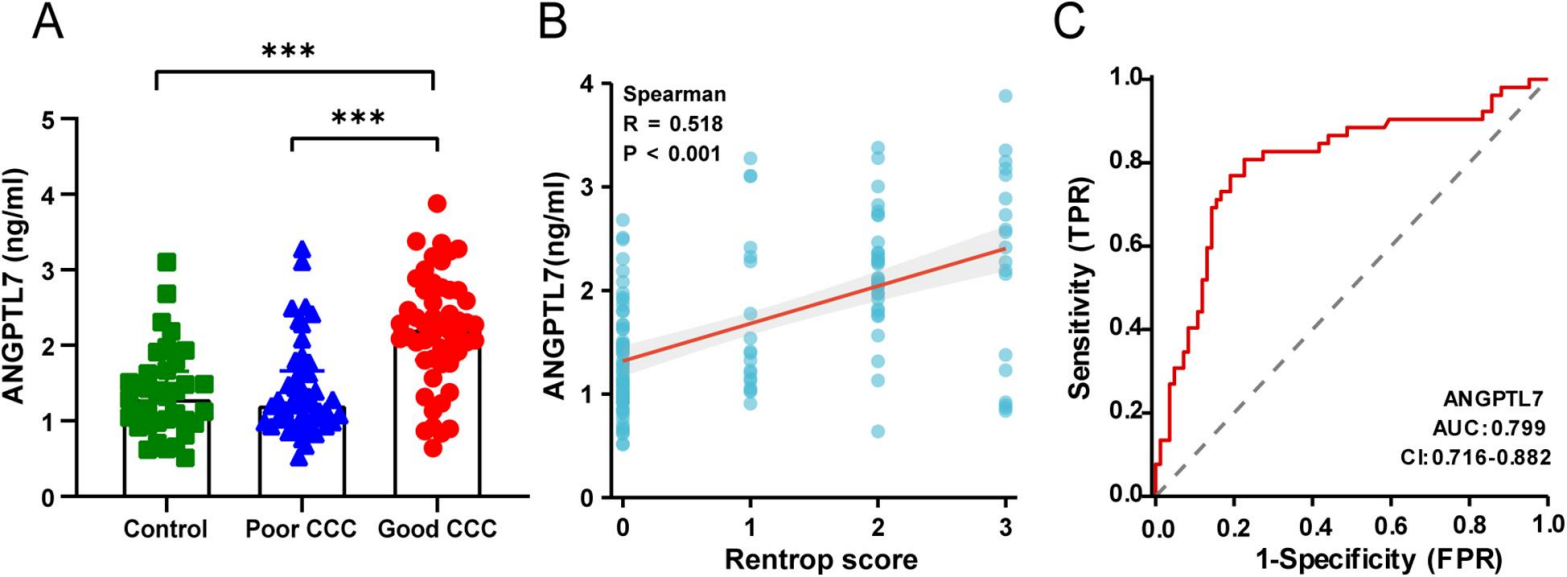
The associations between serum ANGPTL7 levels and CCC. **(A)** Comparison of serum ANGPTL7 levels among three groups. **(B)** Scatter plot of serum ANGPTL7 levels and the Rentrop score. **(C)** ROC curve for serum ANGPTL7 in the prediction of good CCC. CI, confidence interval; ***P < 0.001.

To explore the association between serum ANGPTL7 and CCC, 136 hospitalized patients who underwent CAG at Qilu Hospital of Shandong University for the first time from August 2020 to March 2023 were included. Formation of CCC was predominantly observed in patients with SCS. Thus, patients with diameter stenosis ≥90% (*n* = 100) in at least one main coronary artery were divided into poor collateral circulation group (Rentrop score ≤1, *n* = 48) and good collateral circulation group (Rentrop score >1, *n* = 52), while patients with diameter stenosis <90% in all main coronary arteries comprised the control group (*n* = 36). Baseline characteristics were summarized in Table 1. Versus the control, NT-proBNP levels were significantly elevated in both the good CCC group and the poor CCC group (*P* < 0.05), but with no significant difference observed between these two groups. Ventricular wall segmental motion abnormalities were more prevalent in both the good and the poor CCC group compared to the control group (*P* < 0.05). The incidence of severe RCA stenosis was notably higher in the good CCC group than in the poor group (*P* < 0.05). More patients received coronary revascularization in both the good and the poor CCC group than the control (*P* < 0.05). Additionally, the incidences of MACE were significantly different among the three groups (*P* = 0.005), with the highest proportion observed in the poor CCC group. Most importantly, patients in the control group [1.30 (0.97, 1.65) ng/ml] and the poor CCC group [1.22 (0.98, 1.66) ng/ml] exhibited significantly lower serum ANGPTL7 levels than those in the good CCC group [2.22 (1.81, 2.73) ng/ml] (*P* < 0.001; Figure 3A). There were no significant differences in other baseline variables among groups (*P* > 0.05).

**Table 1:** Baseline characteristics of patients in the control group and with poor/good CCC.

Variables	Control (*n* = 36)	Poor CCC (*n* = 48)	Good CCC (*n* = 52)	*P*-value
General conditions				
Age (years)	60.61 ± 10.12	65.23 ± 8.63	61.81 ± 10.28	0.071
Male, *n* (%)	20 (55.6)	31 (64.6)	40 (76.9)	0.102
BMI (kg/m^2^)	25.55 ± 3.81	25.53 ± 3.11	25.57 ± 3.38	0.998
Hypertension, *n* (%)	23 (63.9)	32 (66.7)	29 (55.8)	0.509
DM, *n* (%)	14 (38.9)	13 (27.1)	24 (46.2)	0.141
Smoking, *n* (%)	7 (19.4)	20 (41.7)	21 (40.4)	0.067
Drinking, *n* (%)	10 (27.8)	21 (43.8)	26 (50.0)	0.110
FH of CHD, *n* (%)	3 (8.3)	9 (18.8)	10 (19.2)	0.329
Carotid plaque, *n* (%)	31 (86.1)	42 (87.5)	51 (98.1)	0.081
Laboratory tests				
NT-proBNP (pg/ml)	79.00 (45.55, 140.28)	144.50 (82.87, 332.75)*	281.95 (121.75, 620.95)*	**<0.001**
hs-cTNI (ng/L)	4.24 (1.95, 7.41)	3.58 (0.01, 8.53)	4.37 (0.01, 13.19)	0.757
SCr (μmol/L)	68.72 ± 13.09	72.02 ± 13.44	75.35 ± 14.15	0.082
ALT (U/L)	19.00 (12.25, 27.00)	19.00 (13.00, 25.75)	19.50 (13.25, 33.25)	0.633
AST (U/L)	18.50 (16.25, 23.50)	19.00 (16.00, 23.00)	19.00 (16.25, 23.00)	0.887
FPG (mmol/L)	5.53 (4.85, 7.09)	5.07 (4.68, 5.91)	5.21 (4.63, 6.02)	0.208
TC (mmol/L)	3.77 ± 0.90	3.74 ± 0.72	3.85 ± 1.16	0.860
LDL-C (mmol/L)	2.16 ± 0.69	2.15 ± 0.57	2.27 ± 0.93	0.698
HDL-C (mmol/L)	1.06 ± 0.25	1.05 ± 0.20	1.03 ± 0.27	0.820
TG (mmol/L)	1.41 (1.00, 1.80)	1.39 (1.03, 1.74)	1.34 (0.93, 1.84)	0.970
Echocardiography				
LVEF	0.65 (0.61, 0.66)	0.61 (0.60, 0.67)	0.61 (0.52, 0.65)	0.066
IVS (mm)	10.00 (9.00, 11.00)	10.50 (10.00, 12.00)	11.00 (10.00, 12.00)	0.085
wall-motion abnormality, *n* (%)	0 (0.0)	14 (29.2)*	25 (48.1)*	**<0.001**
Severe stenosis				
LAD, *n* (%)	–	34 (70.8)	29 (55.8)	0.119
LCX, *n* (%)	–	23 (47.9)	21 (40.4)	0.448
RCA, *n* (%)	–	16 (33.3)	36 (69.2) ^#^	**0.001**
Multivessel, *n* (%)	–	20 (41.7)	29 (55.8)	0.159
Cardiovascular medications				
antiplatelet drugs, *n* (%)	36 (100.0)	48 (100.0)	52 (100.0)	–
statins, *n* (%)	36 (100)	48 (100.0)	52 (100.0)	–
β-blockers, *n* (%)	30 (83.3)	42 (87.5)	44 (84.6)	0.854
ACEI/ARB/ARNI, *n* (%)	13 (36.1)	21 (43.8)	27 (51.9)	0.335
CCB, *n* (%)	8 (22.2)	16 (33.3)	11 (21.2)	0.324
nitrates, *n* (%)	18 (50.0)	19 (39.6)	24 (46.2)	0.619
nicorandil, *n* (%)	25 (69.4)	38 (79.2)	36 (69.2)	0.467
Coronary revascularization, *n* (%)	13 (36.1)	39 (81.3)*	46 (88.5)*	**<0.001**
MACE, *n* (%)	4 (11.1)	18 (37.5)*	8 (15.4)**	**0.005**

Data were presented with mean ± SD, median with interquartile range or *n* (%). BMI, body mass index; DM, diabetes mellitus; FH of CAD, family history of coronary heart disease; NT-proBNP, N-terminal pro-brain natriuretic peptide; hs-cTNI, high-sensitivity cardiac troponin I; SCr, serum creatinine; ALT, alanine aminotransferase; AST, aspartate transaminase; FPG, fasting plasma glucose; TC, total cholesterol; LDL-C, low-density lipoprotein-cholesterol; HDL-C, high-density lipoprotein-cholesterol; TG, triglyceride; LVEF, left ventricle ejection fraction; IVS, intraventricular septum; ACEI, angiotensin-converting enzyme inhibitors; ARB, angiotensin receptor blockers; ARNI, angiotensin receptor & neprilysin inhibitors; CCB, calcium channel blockers; SD, standard deviation. *P* values in bold are <0.05. Symbols denote significant differences compared to the control group.

* *P* < 0.05) or the poor CCC group.

** *P* < 0.05).

Given the variation in serum ANGPTL7 levels among different CCC groups, a correlation and predictive analysis was carried out. Spearman correlation analysis indicated a significantly positive correlation between serum ANGPTL7 and the Rentrop score (*r* = 0.518, *P* < 0.001; Figure 3B). Receiver-operating characteristics (ROC) curve analysis revealed that the area under the curve (AUC) for serum ANGPTL7 in predicting good CCC was 0.799 (95% CI: 0.716–0.882, *P* < 0.001; Figure 3C), with the optimal cut-off value at 1.712, showing a sensitivity of 80.77% and specificity of 77.38%. These findings collectively suggest a close asssociation between serum ANGPTL7 levels and CCC.

### 3.3 Serum ANGPTL7 remarkably increased in CHD patients with SCS and MI mice

**Figure 4:**
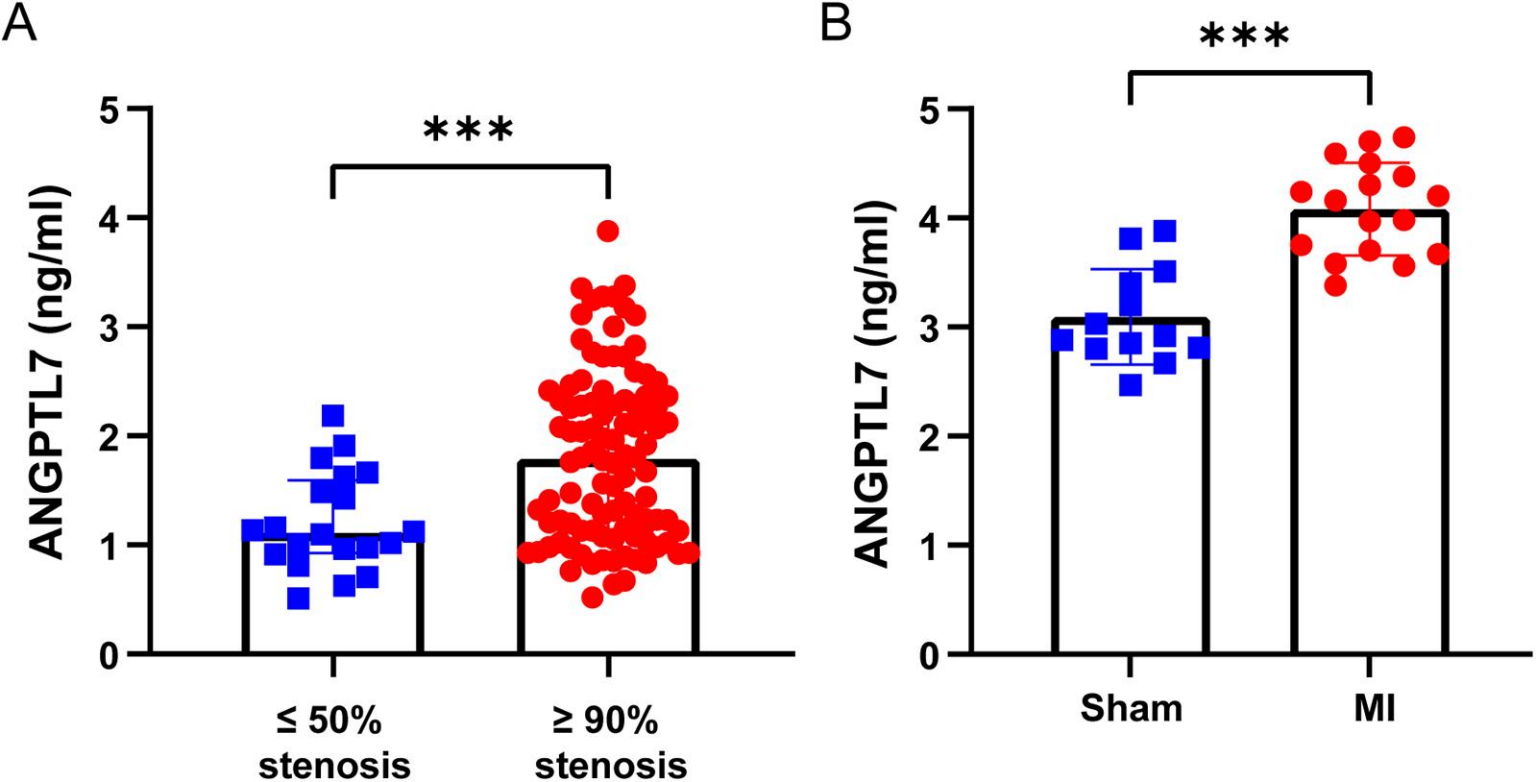
Elevated serum ANGPTL7 in both patients with SCS and post-infarct mice. **(A)** Comparison of serum ANGPTL7 levels of patients with ≤50% coronary stenosis and ≥90% stenosis by ELISA. **(B)** Comparison of serum ANGPTL7 levels of mice in the sham and the MI group by ELISA. ***P < 0.001.

In our study, the formation of coronary collaterals was mainly observed to be concomitant with coronary severe stenosis or even occlusion. Hence, we investigated the change of serum ANGPTL7 level in CHD patients with SCS and mice after MI surgery by ELISA. As presented in Figure 4A, patients with ≥90% coronary stenosis had significantly higher levels of serum ANGPTL7 [1.79(1.13, 2.35) ng/ml] than those with ≤50% stenosis [1.11(0.93, 1.59) ng/ml] with a significantly statistical difference (*P* < 0.001). Besides, there was a remarkable elevation of serum ANGPTL7 for mice in the MI group (4.08 ± 0.43 ng/ml) compared to the control group (3.09 ± 0.44 ng/ml) with a significant difference (*P* < 0.001; Figure 4B). These findings suggest a potential relationship between serum ANGPTL7 and CHD characterized by SCS.

### 3.4 Serum ANGPTL7 performed a prognostic value for MACE in patients with SCS

**Figure 5:**
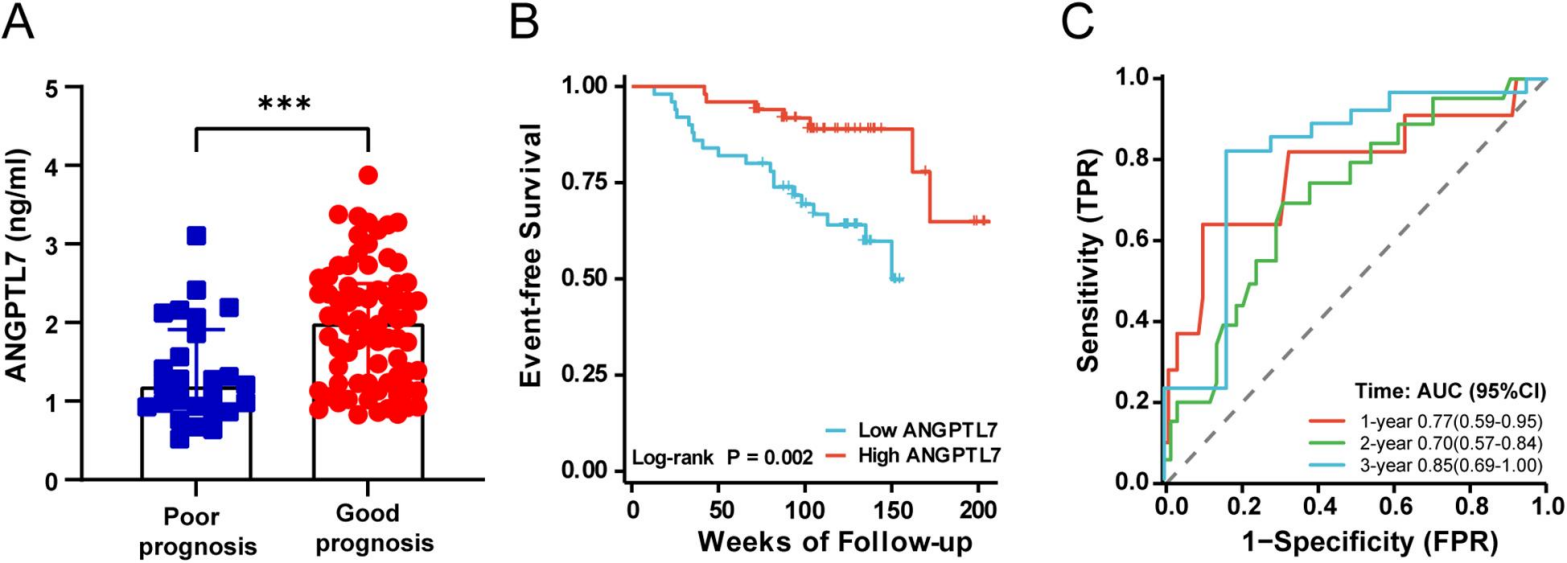
The prognostic performance of serum ANGPTL7 for patients with SCS. **(A)** Comparison of serum ANGPTL7 levels of patients in the poor and the good prognosis group. **(B)** Kaplan–Meier survival curves for MACE between different levels of serum ANGPTL7 according to the median concentration. **(C)** Time-dependent ROC curves of serum ANGPTL7 for the prediction of MACE. AUC, area under the curve; CI, confidence interval; ***P < 0.001.

To investigate whether there was an association between serum ANGPTL7 levels and the prognosis of CHD patients with SCS, 100 patients with ≥90% coronary stenosis were retrospectively followed up for 13–207 weeks. The total number of MACE cases was 26 (26.0%), including 2 (2.0%) cases of all-cause death, 1 (1.0%) case of cardiac death, 6 (6.0%) cases of non-fatal MI, 12 (12.0%) cases of coronary revascularization and 5 (2.8%) cases of non-fatal stroke, which consisted of the poor prognosis group. The serum concentrations of ANGPTL7 in the good and the poor prognosis group were [2.01(1.23, 2.50)] ng/ml and [1.20(0.91, 1.91)] ng/ml, respectively, with a significantly statistical difference (*P* < 0.001; Figure 5A). Moreover, a Kaplan–Meier survival plot was generated to display the outcomes of patients stratified based on serum ANGPTL7 levels around the median concentration and showed a significantly lower cumulative incidence of MACE for the high ANGPTL7 group (Figure 5B). Table 2 showed that the serum ANGPTL7 level, whether as a categorical or continuous variable, was significantly associated with MACE on univariable Cox proportional hazards regression analysis (Per SD increase: HR = 0.351, 95% CI: 0.196–0.628, *P* < 0.001; High ANGPTL7 group: HR = 0.221, 95% CI: 0.082–0.592, *P* = 0.003). It remained a protective factor after fully adjusting for covariates on multivariable Cox regression analysis in Model 4 (Per SD increase: HR = 0.328, 95% CI: 0.152–0.710, *P* *=* 0.005; High ANGPTL7 group: HR = 0.277, 95% CI: 0.079–0.964, *P* = 0.044). To further evaluate the prognostic value of ANGPTL7, time-dependent ROC curve analysis was performed and the AUC reached 0.77 at 1 year, 0.70 at 2 years and 0.85 at 3 years (Figure 5C).

**Table 2:** Association of serum ANGPTL7 level and MACE via Cox regression analyses.

ANGPTL7	Model1 [HR(95%CI]	*P* value	Model2 [HR(95%CI]	*P* value	Model3 [HR(95%CI]	*P* value	Model4 [HR(95%CI]	*P* value
*MACE*								
Per 1 SD increase	0.351 (0.196–0.628)	<0.001	0.337 (0.187–0.607)	<0.001	0.395 (0.197–0.788)	0.008	0.328 (0.152–0.710)	0.005
High ANGPTL7 group	0.221 (0.082–0.592)	0.003	0.217 (0.081–0.582)	0.002	0.308 (0.101–0.938)	0.038	0.277 (0.079–0.964)	0.044

CI, confidence interval; HR, hazard ratio; SD, standard deviation; MACE, major adverse cardiovascular events; ANGPTL7, angiopoietin-like protein 7; LAD, left anterior descending artery; LCX, left circumflex artery; NT-proBNP, N-terminal pro-brain natriuretic peptide; DM, diabetes mellitus; LDL-C, low-density lipoprotein cholesterol; ACEI, angiotensin-converting enzyme inhibitor; ARB, angiotensin receptor blocker; ARNI, angiotensin receptor-neprilysin inhibitor.

Model 1: no covariates were adjusted for.

Model 2: adjusted for age and gender.

Model 3: adjusted for age, gender and all the variables significant on univariate analysis (including Rentrop score, LAD and LCX severe stenosis, hypertension and NT-proBNP).

Model 4: adjusted for age, gender, hypertension, DM, NT-proBNP, LDL-C, Rentrop score, LAD and LCX severe stenosis, wall-motion abnormality, the usage of β-blocker and ACEI/ARB/ARNI.

### 3.5 ANGPTL7 promoted angiogenesis *in vitro*

**Figure 6:**
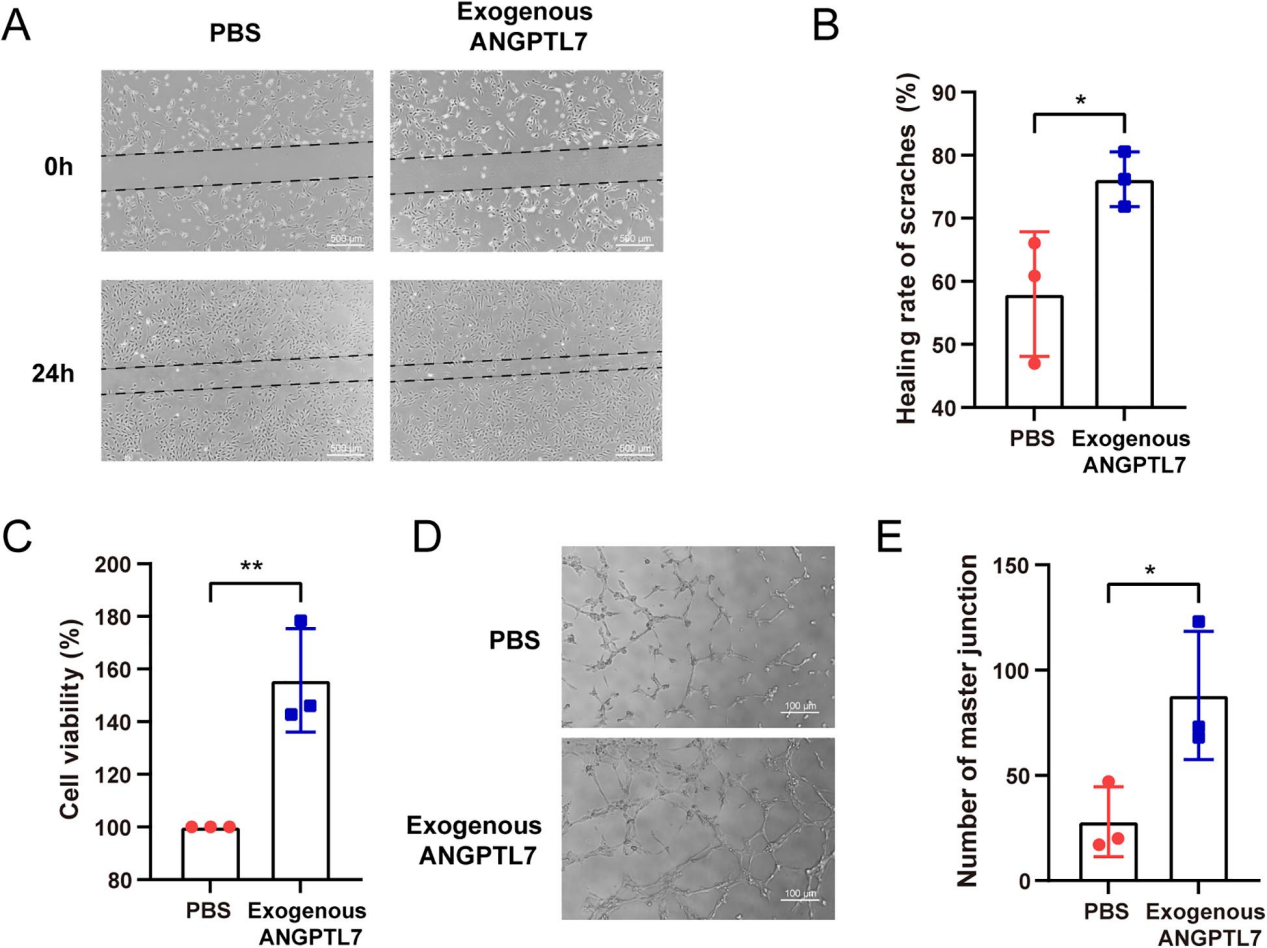
The enhanced endothelial cell proliferation, migration and capillary-like structure formation induced by exogenous ANGPTL7. **(A)** Representative images of scratch assays (40× magnification, scan bar = 500 μm). **(B)** Comparison of scratch healing rates [=(0 h scratch width-24 h scratch width)/0 h scratch width × 100%] between the exogenous ANGPTL7 group and PBS group (three independent experiments were performed, *P < 0.05). **(C)** Cell viabilities (=the relative ratio of cell number) in CCK8 assays were compared between the two groups (three independent experiments were performed, **P < 0.01). **(D)** Representative images of tube formation assays (10× magnification, scan bar = 100 μm). **(E)** Number of master junctions was calculated and compared between the two groups (three independent experiments were performed, *P < 0.05).

**Figure 7:**
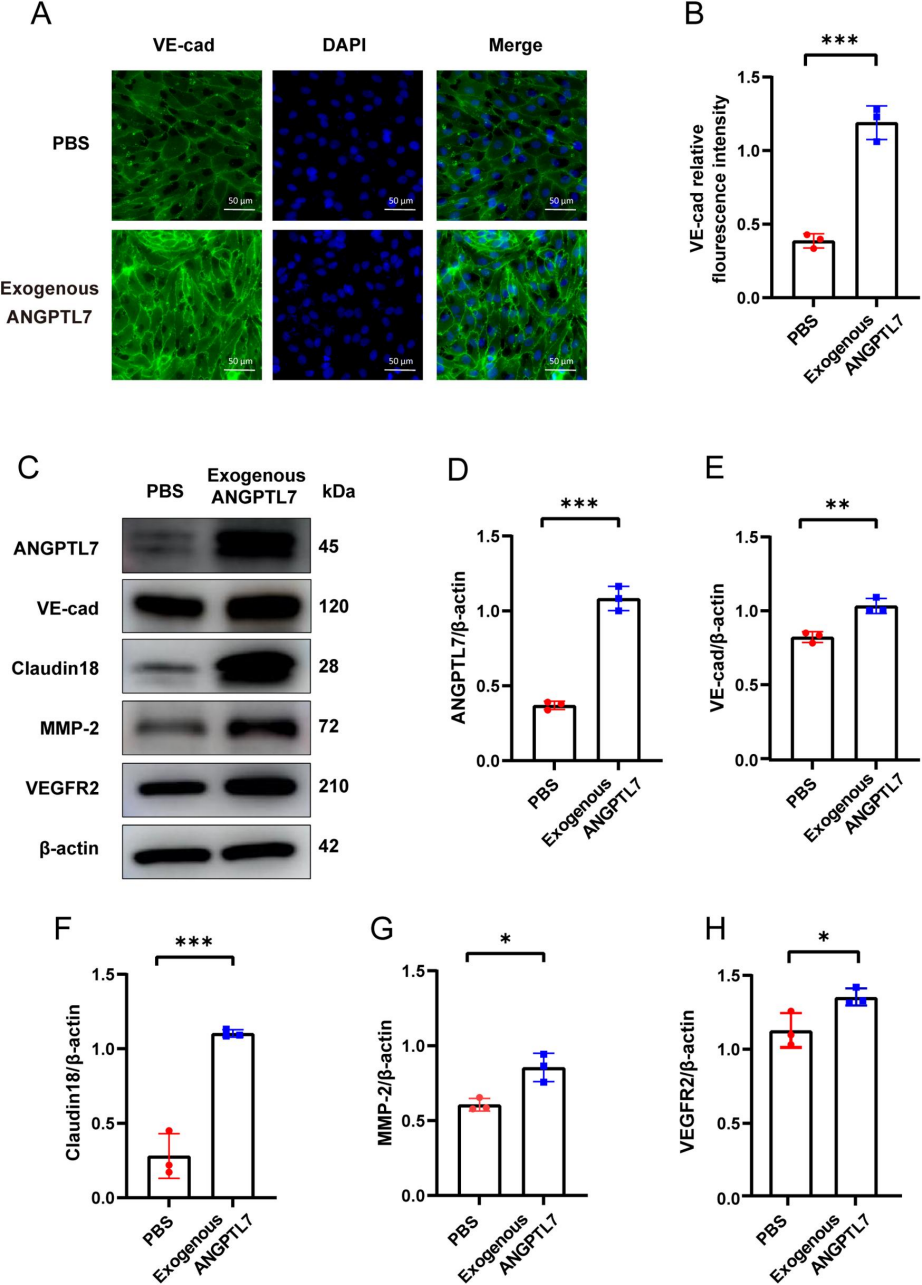
The proangiogenic property of ANGPTL7. **(A)** Representative images of cell immunofluorescence assays (40× magnification, scan bar = 50 μm, VE-cadherin expression region exhibits green fluorescence). **(B)** VE-cadherin expression was detected and quantified by immunofluorescence after 24-h stimulation by exogenous human recombinant ANGPTL7 protein, compared with PBS as control (three independent experiments were performed, ***P < 0.001). **(C)** Representative images of western blot detecting protein levels of ANGPTL7, VE-cadherin, Claudin18, MMP-2, VEGFR2 and internal control β-actin in HUVECs. Western blot analysis was performed to compare the protein levels of ANGPTL7 **(D)**, VE-cadherin **(E)**, Claudin18 **(F)**, MMP-2 **(G)** and VEGFR2 **(H)** between the two groups (three independent experiments were performed, *P < 0.05, **P < 0.01, ***P < 0.001).

To evaluate the angiogenic impact of ANGPTL7, we treated HUVECs with exogenous human recombinant ANGPTL7 protein or PBS as control. The scratch assay revealed a substantial enhancement in the migratory ability of HUVECs following stimulation by recombinant ANGPTL7 (Figures 6A,B). Moreover, the CCK8 assay denmonstrated that ANGPTL7 facilitated the proliferation of vascular endothelial cells (Figure 6C). Furthermore, the tube formation assay illustrated the superior tube-forming capacity of endothelial cells in the experimental group (Figures 6D,E). Vascular endothelial cadherin (VE-cadherin) is a prominent adhesive protein of endothelial adherens junctions (24). Cell immunofluorescence assays revealed a notable elevation in VE-cadherin expression in the experimental group compared to the PBS group, with a statistically significant difference (Figures 7A,B), which means HUVECs adhere more strongly to form neovessels after adding exogenous human recombinant ANGPTL7 protein as a stimulant.Claudin18 is an important component of endothelial tight junctions (25). Matrix metalloproteinase-2 (MMP-2) is an extensively-investigated member of MMPs, which can contribute to angiogenic process through the degradation of extracellular matrix barrier (26). Vascular endothelial growth factor A (VEGF) and its receptor VEGFR2 represent a key signalling pathway mediating angiogenesis (27). Subsequent western blot results showed that the protein levels of VE-cadherin, Claudin18, MMP-2 and VEGFR2 in HUVECs all significantly increased after treatment with exogenous human recombinant ANGPTL7 (Figures 7C–H). In aggregrate, these findings provide initial evidence *in vitro* indicating the proangiogenic effect of ANGPTL7, including the promotion of vessel maturation and stability during angiogenesis.

To further interrogate the possible mechanisms by which ANGPTL7 promoted angiogenesis, we identified enriched KEGG pathways using GSEA. Top 10 of the pathway enrichment results were shown in Supplementary Figure 1, which can provide initial references for the study on downstream mechanisms in the future.

## 4 Discussion

This study represents a pioneering investigation into the association among ANGPTL7, angiogenesis, CCC, as well as clinical prognosis of CHD patients with SCS. The findings indicate that serum ANGPTL7 exhibits robust specificity and sensitivity in forecasting the extent of CCC, thereby suggesting its potential value as a prognostic biomarker for patients with SCS. Additionally, both mouse model experiments and *in vitro* studies provided further evidence supporting the role of ANGPTL7 in promoting angiogenesis in response to myocardial ischemia following SCS or total occlusion.

Numerous studies on CHD patients have reported lots of clinical factors in relation to the formation of CCC, such as bradycardia (28), exercise (29), metabolic syndrome (including abdominal obesity, hyperglycemia, hypertension, hypertriglyceridemia and low HDL-C) (30), aging (31), smoking, alcoholism (32), the severity of coronary artery lesions (33) and so on. Our study found a significant relationship between severe stenosis of the RCA and CCC levels. The incidence of RCA severe stenosis was notably higher in the good CCC group compared to the poor group, aligning with conclusions from other similar studies. Past researches also indicated that in patients with coronary artery occlusion, those with RCA occlusion tended to exhibit better coronary collateral circulation (34, 35). This possibly ascribe to the fact that LAD and LCX can provide double compensatory collateral supply after severe RCA stenosis or occlusion. Moreover, significant disparities were observed in NT-proBNP, ventricular wall segmental motion abnormalities and acceptance of revascularization between the control group and the other two groups, but no statistical significant differences were found between the good and the poor CCC group. Consequently, the three variables mentioned above were not identified as factors associated with CCC grades in our study. Up till now, studies on the relationship between CCC and the prognosis of CHD have been conducted in patients with acute MI (36), ischemic cardiomyopathy, stable coronary artery disease (37), chronic total occlusion (38), etc. Although a small number of studies suggest no correlation with a reduced risk of MACE (39), the prevailing consensus among scholars is that good CCC brings about a better clinical prognosis. Our study's findings revealed a significantly lower incidence of MACE in the good CCC group compared to the poor group after retrospective follow-up, supporting the mainstream view.

Previous studies once suggested that the formation of CCC is not a process of angiogenesis but solely a result of arteriogenesis (40). That is, the remodeling of pre-existing arterioles gives rise to the lumen enlargement and tube wall thickening, which causes a 10–20 fold increase in blood flow to compensate for the reduced blood supply of occluded vessels (41). But recently, a growing number of studies have highlighted that angiogenesis, as an early step of arteriogenesis, plays a critical role in the formation of CCC. According to a murine model study about coronary collateral growth, researchers induced repetitive myocardial ischemia by placing a pneumatic occluder over the left coronary artery of mice. They found the expression of angiogenesis-related genes, like Cxcr4, Vegfr2, Jag1, Mcp1, and Hif1α, were upregulated at the early stage of the repetitive ischemia-induced coronary collateral growth, indicating the involvement of sprouting endothelial cells in capillary angiogenesis (4), This angiogenic process results in the development of new capillaries extending into functional coronary collateral branches. Myocardial ischemia is now considered to be one of the crucial factors affecting CCC formation, particularly at the early stage (42, 43), where it promotes the secretion of pro-angiogenic factors (44). Yu et al. has revealed the role of interleukin-33 (IL-33) in angiogenesis and cardiac repair after MI for protecting cardiac ischemic injury (45). The methods of this study has provided an important reference to us. Similar results has been found in our study about the proangiogenic effect of ANGPTL7. Subsequently, the newly formed vessels mature into the established coronary collateral circulation, enhancing blood supply to ischemic regions.

Recent researches have shed light on the controversial effects of ANGPTL7 on angiogenesis. Despite being initially thought to possess antiangiogenic properties due to its expression in cornea (14), latest studies have challenged this perception. Parri et al. conducted a study demonstrating that ANGPTL7 is overexpressed in human colorectal cancer and exerts a proangiogenetic effect on HUVECs. They found that ANGPTL7 enhances proliferation, migration, invasiveness and the capacity to form capillary-like vascular lumens of endothelial cells. In addition, they observed that ANGPTL7 promotes neovascularization *in vivo* using mouse Matrigel sponge assay (16). In our opinion, the varying conclusions regarding the effects of ANGPTL7 on angiogenesis may be attributed to the differences in experimental methodologies. The studies supporting the proangiogenetic effect of ANGPTL7 directly utilized recombinant ANGPTL7 protein to stimulate HUVECs and validated the findings with mouse models (16). Conversely, studies indicating antiangiogenic properties of ANGPTL7 relied on the culture medium supernatant from ANGPTL7-overexpressed tumor cells (46) or corneal cell co-cultures (18), which could be easily interferred by other substances secreted by tumor or corneal stromal cells. What's more, tissue-specific effects should not be overlooked. ANGPTL7 is an anti-angiogenic factor in human cornea and responsible for maintaining tissue avascularity. However, in vascular endothelial cells of tumor, it may regulate pathways of angiogenic sprouting and microvascular remodeling through the interaction with microenvironmental factors. To address this discrepancy, we employed exogenous human ANGPTL7 recombinant protein as a stimulant for HUVECs and found enhanced migration, proliferation, tube formation, vessel maturation and stability, basically aligning with the results of Parri et al. (16).

Current studies only reported the angiogenic effect of ANGPTL7, however, the association between serum ANGPTL7 and CCC has not yet been explored. Our study reported for the first time that serum ANGPTL7 level was positively associated with increased CCC grades. For patients with severe coronary stenosis, participants in the control group and the poor CCC group both had lower serum ANGPTL7 concentrations than the good CCC group. Furthermore, ROC curve analysis showed serum ANGPTL7 had a good predictive performance for CCC levels. Evidence from mouse studies suggests that neocollaterals form rapidly after coronary occlusion in mice (47). It was also found in our sudy that the mice in MI group had significantly higher serum ANGPTL7 levels as determined by ELISA. Taken together, ANGPTL7 appears to be intimately associated with the formation of CCC by influencing angiogenesis.

There have been relatively few studies on the role of ANGPTL7 in cardiovascular diseases. The serum level of ANGPTL7 was reported to be independently associated with the short-term mortality among patients with acute heart failure (48), but it remained to be further explored whether it is involved in myocardial remodeling. Moreover, ANGPTL7 seemed to have relavence to high-risk factors of atherosclerosis and CHD such as inflammation (49), endothelial cell injury (50), obesity (51) and diabetes (52). Therefore, further investigations into the role of ANGPTL7 in the development of CHD are warranted. Currently, there is no available data on the correlation between ANGPTL7 and the prognosis of CHD patients with SCS, which is defined as ≥90% stenosis in at least one major coronary artery. Our study revealed for the first time that the patients with MACE had significantly lower levels of serum ANGPTL7 than those without events after about 4-year follow-up. Through Cox proportional hazards regression analysis and time-dependent ROC curve analysis, we finally demonstrated that ANGPTL7 is an independent prognostic factor with a good predictive value for MACE in patients with SCS. Combining the clinical findings with *in vitro* studies, it is resonable to draw an inference that the impact of ANGPTL7 on CHD outcomes probably attributed to its role in CCC. However, this remains a speculative hypothesis by now and requires further investigations.

The exciting translational potential of the promising prognostic value of ANGPTL7 in patients with SCS warranted a discussion on its possible integration into clinical practice. Firstly, the quantitative assessment of serum ANGPTL7 could augment current risk stratification models. By providing an objective measure of the angiogenic capacity, it may help identify a subgroup of patients with poor CCC development who are at heightened risk for future MACE, thus meriting more aggressive therapeutic interventions or closer follow-up. Secondly, serial measurement of ANGPTL7 levels could serve as a dynamic biomarker to monitor therapeutic efficacy. For instance, it could be used to assess the response to treatments of promoting angiogenesis or to evaluate the natural progression of collateralization over time. Finally, with the development of therapies targeting angiogenesis, pretreatment ANGPTL7 levels might help select patients who are most likely to benefit from such proangiogenic therapies, paving the way for a more personalized approach in managing CHD. While these applications are speculative, they are grounded in the strong predictive value we observed and mirror the clinical development of other cardiovascular biomarkers.

Several limitations of our study should be considered. First, this was a single-center retrospective study with a relatively small sample size, which may cause selectivity bias, so negative findings in our study should be interpreted cautiously and large-scale and prospective studies were essential in the future. Second, we adopted the Rentrop score system, the most routinely used approach, to evaluate the presence and degree of CCC. Yet this is a relatively subjective method and other assessments like CFI may be more accurate. Third, although HUVECs are a standard model for endothelial studies, the use of cardiac-specific endothelial cells would enhance the translational relevance of the findings to CCC. Therefore, primary cardiac endothelial cells or a cardiac microvascular endothelial cell line were considerable in further studies. Finally, our *in vitro* experiments only demonstrated the proangiogenic effect of ANGPTL7, but the study on the exploration of its downstream mechanism in coronary collateral formation was inadequate.

## 5 Conclusion

In conclusion, this study revealed the association among ANGPTL7, angiogenesis, CCC and the prognosis of patients with SCS. The serum level of ANGPTL7 was positively correlated to CCC and could serve as a biomarker for evaluating the coronary collateral level, which is probably attributed to its ability to promote collateral angiogenesis. In addition, the serum ANGPTL7 has a potential predictive value for the prognosis of CHD patients with SCS. Specifically, it was concluded that a higher serum level of ANGPTL7 was associated with a better clinical outcome. Finally, ANGPTL7 performed a proangiogenic effect on vascular endothelial cells.

## Data Availability

The original contributions presented in the study are publicly available. This data can be found here: https://www.ncbi.nlm.nih.gov/geo/query/acc.cgi?acc=GSE308783.
