# Supplementary material for: Associations of angiopoietin-like protein 7 with coronary collateral circulation and prognosis of patients with severe coronary artery stenosis
Source: Front Cardiovasc Med. 2025 Oct 10;12:1559267. doi: 10.3389/fcvm.2025.1559267 (PMC12550588; doi:10.3389/fcvm.2025.1559267)
Supplement: Supplementary file 1 [file Datasheet1.pdf]

Supplementary Material

1 Supplementary Tables

Table S1 Baseline characteristics of all the patients in control group

Variables	Lost to follow up (n=19)	Finally included (n=36)	*P*-value
General conditions			
Age (years)	62.74±4.69	60.61±10.12	0.293
Male, n (%)	12 (63.2)	20 (55.6)	0.587
BMI (kg/m^2)	25.43±2.26	25.55±3.81	0.905
Hypertension, n (%)	10 (52.6)	23 (63.9)	0.418
DM, n (%)	6 (31.6)	14 (38.9)	0.592
Smoking, n (%)	5 (26.3)	7 (14.9)	0.808
Drinking, n (%)	8 (42.1)	10 (27.8)	0.282
FH of CHD, n (%)	3 (15.8)	3 (8.3)	0.698
Carotid plaque, n (%)	16 (84.2)	31 (86.1)	1.000
Laboratory tests			

NT-proBNP (pg/mL)	110.00 (75.00,256.00)	79.00 (45.55,140.28)	0.144
hs-cTNI (ng/L)	3.53 (1.92,6.11)	4.24 (1.95,7.41)	0.723
SCr (μmol/L)	69.63±11.67	68.72±13.09	0.801
ALT (U/L)	20.16±13.61	20.56±10.24	0.091
AST (U/L)	21.00 (16.00,23.00)	18.50 (16.25,23.50)	0.710
FPG (mmol/L)	5.68 (4.12,6.42)	5.53 (4.85,7.09)	0.426
TC (mmol/L)	4.17 (3.25,4.47)	3.63 (3.22,4.25)	0.344
LDL-C (mmol/L)	2.40±0.46	2.16±0.69	0.168
HDL-C (mmol/L)	1.05±0.33	1.06±0.25	0.887
TG (mmol/L)	1.48 (1.03,1.92)	1.41 (1.00,1.80)	0.626
Echocardiography			
LVEF	0.62±0.05	0.64±0.04	0.095
IVS (mm)	10.00 (9.00,11.00)	10.00 (9.00,11.00)	0.269
Cardiovascular medications			
antiplatelet drugs, n (%)	19 (100.0)	36 (100.0)	——

statins, n (%)	19 (100.0)	36 (100.0)	——
β-blockers, n (%)	14 (73.7)	30 (83.3)	0.620
ACEI/ARB/ARNI, n (%)	7 (36.8)	13 (36.1)	0.957
CCB, n (%)	7 (36.8)	8 (22.2)	0.247
nitrates, n (%)	8 (42.1)	18 (50.0)	0.577
nicorandil, n (%)	13 (68.4)	25 (69.4)	0.938
Coronary revascularization, n (%)	7 (36.8)	13 (36.1)	0.957

Data were presented with mean±SD, median with interquartile range or n(%). BMI, body mass index; DM, diabetes mellitus; FH of CAD, family history of coronary heart disease; NT-proBNP, N-terminal pro-brain natriuretic peptide; hs-cTNI, high-sensitivity cardiac troponin I; SCr, serum creatinine; ALT, alanine aminotransferase; AST, aspartate transaminase; FPG, fasting plasma glucose; TC, total cholesterol; LDL-C, low-density lipoprotein-cholesterol; HDL-C, high-density lipoprotein-cholesterol; TG, triglyceride; LVEF, left ventricle ejection fraction; IVS, intraventricular septum; ACEI, angiotensin-converting enzyme inhibitors; ARB, angiotensin receptor blockers; ARNI, angiotensin receptor & neprilysin inhibitors; CCB, calcium channel blockers; SD, standard deviation.

Table S2 Baseline characteristics of all the patients in SCS group

Variables	Lost to follow up (n=38)	Finally included (n=100)	*P*-value
General conditions			
Age (years)	63.34±6.47	63.45±9.63	0.940
Male, n (%)	26 (68.4)	71 (71.0)	0.767
BMI (kg/m^2)	26.27±2.15	25.55±3.24	0.137
Hypertension, n (%)	25 (65.8)	61 (61.0)	0.604
DM, n (%)	16 (42.1)	37 (37.0)	0.582
Smoking, n (%)	16 (42.1)	41 (41.0)	0.906
Drinking, n (%)	16 (42.1)	47 (47.0)	0.606
FH of CHD, n (%)	7 (18.4)	19 (19.0)	0.938
Carotid plaque, n (%)	34 (89.5)	93 (93.0)	0.740
Laboratory tests			
NT-proBNP (pg/mL)	142.60 (57.25,655.00)	199.90 (108.48,448.75)	0.158
hs-cTNI (ng/L)	7.24 (3.34,20.66)	4.17 (0.01,10.69)	0.074

SCr (μmol/L)	73.11±13.36	73.75±13.85	0.806
ALT (U/L)	27.50 (12.75,42.25)	19.00 (13.00,28.00)	0.204
AST (U/L)	21.50 (16.75,26.50)	19.00 (16.00,23.00)	0.146
FPG (mmol/L)	5.70 (4.53,6.32)	5.15 (4.68,6.00)	0.291
TC (mmol/L)	3.85±0.51	3.80±0.97	0.676
LDL-C (mmol/L)	2.17±0.47	2.21±0.77	0.728
HDL-C (mmol/L)	1.03±0.26	1.04±0.24	0.890
TG (mmol/L)	1.49 (1.01,1.89)	1.37 (1.00,1.77)	0.436
Echocardiography			
LVEF	0.60 (0.52,0.64)	0.61 (0.56,0.67)	0.120
IVS (mm)	10.74±1.59	10.90±1.43	0.563
wall-motion abnormality, n (%)	16 (42.1)	39 (39.0)	0.739
Severe stenosis			
LAD, n (%)	24 (63.2)	63 (63.0)	0.986
LCX, n (%)	16 (42.1)	44 (44.0)	0.841
RCA, n (%)	20 (52.6)	52 (52.0)	0.947

Multivessel, n (%)	19 (50.0)	49 (49.0)	0.916
Cardiovascular medications			
antiplatelet drugs, n (%)	38 (100.0)	100 (100.0)	——
statins, n (%)	38 (100.0)	100 (100.0)	——
β-blockers, n (%)	34 (89.5)	86 (86.0)	0.796
ACEI/ARB/ARNI, n (%)	19 (50.0)	48 (48.0)	0.834
CCB, n (%)	8 (21.1)	27 (27.0)	0.473
nitrates, n (%)	17 (44.7)	43 (43.0)	0.854
nicorandil, n (%)	28 (73.7)	74 (74.0)	0.970
Coronary revascularization, n (%)	34 (89.5)	85 (85.0)	0.496

Data were presented with mean±SD, median with interquartile range or n(%). BMI, body mass index; DM, diabetes mellitus; FH of CAD, family history of coronary heart disease; NT-proBNP, N-terminal pro-brain natriuretic peptide; hs-cTNI, high-sensitivity cardiac troponin I; SCr, serum creatinine; ALT, alanine aminotransferase; AST, aspartate transaminase; FPG, fasting plasma glucose; TC, total cholesterol; LDL-C, low-density lipoprotein-cholesterol; HDL-C, high-density lipoprotein-cholesterol; TG, triglyceride; LVEF, left ventricle ejection fraction; IVS, intraventricular septum; ACEI, angiotensin-converting enzyme inhibitors; ARB, angiotensin receptor blockers; ARNI, angiotensin receptor & neprilysin inhibitors; CCB, calcium channel blockers; SD, standard deviation.

2 Supplementary Figures

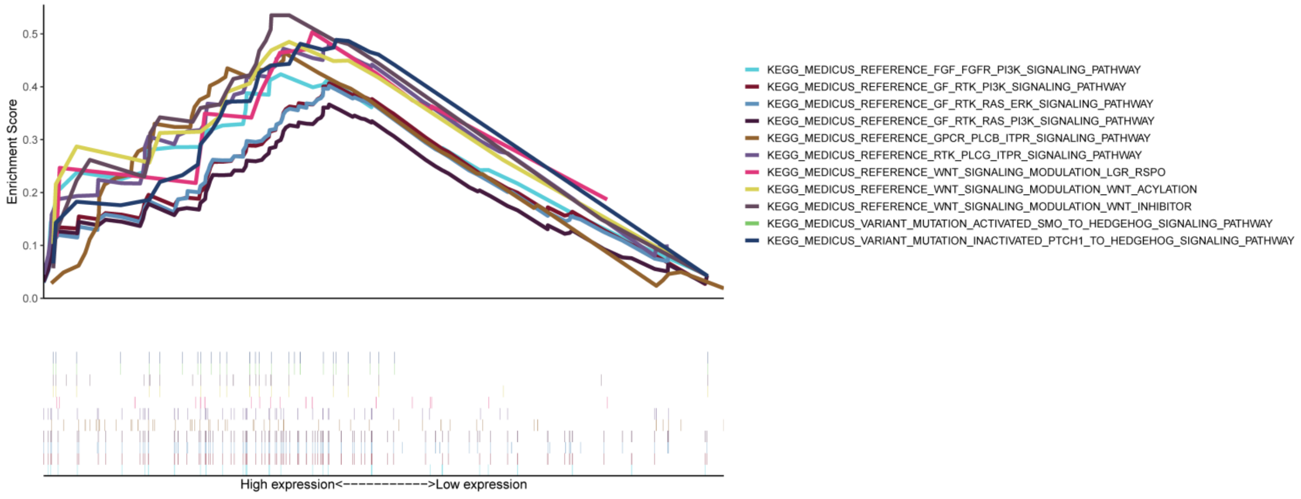

Fig. S1 KEGG pathway enrichment analysis on downstream mechanisms by which ANGPTL7 promotes angiogenesis.
